# Weight Bias Internalization Is Negatively Associated With Weight-Related Quality of Life in Persons Seeking Weight Loss

**DOI:** 10.3389/fpsyg.2018.02576

**Published:** 2018-12-17

**Authors:** Olivia A. Walsh, Thomas A. Wadden, Jena Shaw Tronieri, Ariana M. Chao, Rebecca L. Pearl

**Affiliations:** ^1^Center for Weight and Eating Disorders, Department of Psychiatry, Perelman School of Medicine at the University of Pennsylvania, Philadelphia, PA, United States; ^2^Department of Biobehavioral Health Sciences, School of Nursing, University of Pennsylvania, Philadelphia, PA, United States

**Keywords:** depression, obesity, weight-related quality of life, weight, weight bias internalization

## Abstract

Research has shown a negative relationship between weight bias internalization (WBI) and general measures of health-related quality of life (QOL), such as the Short Form–36. Less is known about the impact of WBI on weight-specific domains of QOL. This study examined the relationship between WBI and weight-related QOL, as measured by the Impact of Weight on Quality of Life (IWQOL-Lite) scale. Participants were 178 adults with obesity [71.3% black, 87.6% female, mean body mass index (BMI) = 40.9 ± 5.9 kg/m^2^] enrolled in a weight loss trial testing the effects of lorcaserin on weight loss maintenance. At baseline, participants completed the Weight Bias Internalization Scale (WBIS), the IWQOL-Lite and the Patient Health Questionnaire (PHQ-9, to assess symptoms of depression). Total scores for the IWQOL-Lite and its five subscales (Physical Function, Self-Esteem, Sexual Life, Public Distress and Work) were calculated. Linear regression analyses showed that WBIS scores were associated with the IWQOL-Lite total score and all subscales above and beyond the effects of demographic variables, BMI, and depressive symptoms (beta values = -0.18 to -0.70, *p* values < 0.019). The relationship between WBIS and the IWQOL-Lite scales did not differ by gender or race. WBI was associated with mental and physical aspects of weight-related QOL in a predominantly black and female treatment-seeking sample of patients with obesity. Prioritizing the development of interventions to reduce WBI may be important for improving weight-related QOL.

## Introduction

Health care professionals, employers, co-workers, parents, passing strangers, and even young children have been found to hold negative attitudes towards persons with overweight and obesity, describing them, for example, as lazy, worthless, awkward, ugly, and lacking in self-esteem (Puhl and Brownell, 2001). These biased attitudes, and the resulting societal devaluation of individuals with overweight and obesity in society (i.e., stigmatization), may be accompanied by discrimination, in which people are treated unfairly in educational, employment, or social settings due to their weight (Puhl and Brownell, 2001). A substantial literature, for example, suggests that both men and women with obesity are paid less than their average-weight counterparts when doing the same work (Baum and Ford, 2004; Cawley, 2004; Puhl and Heuer, 2009).

Some persons with overweight and obesity report sharing society’s biased attitudes toward excess weight, believing that they, in fact, are lazy, undisciplined, or otherwise undesirable because of their weight (Rudman et al., 2002). This form of self-criticism is referred to as weight bias internalization (WBI), or self-directed stigma (Durso and Latner, 2008). A significant minority of persons with overweight or obesity report elevated levels of WBI, with higher levels of WBI among individuals seeking treatment for or engaged in weight loss (Puhl et al., 2018). Those with WBI report significantly greater symptoms of depression, anxiety, and disordered eating than their weight-matched counterparts without WBI (Pearl and Puhl, 2018). WBI is also associated with poor health behaviors (such as avoidance of physical activity) and with increased cardiometabolic risk (Pearl and Puhl, 2018). Prior research also has suggested that individuals with WBI report reduced quality of life (QOL) in both their physical function and mental health, as assessed by general measures such as the Medical Outcome Survey, Short-Form-36 (SF-36) (Latner et al., 2014; Pearl et al., 2014). The SF-36 has been used to assess QOL in a variety of different populations, but it was not constructed to assess impairments in function potentially related to excess body weight. Prior work has shown a relationship between WBI and negative general mental health outcomes (Latner et al., 2013, 2014; Pearl et al., 2014; Hübner et al., 2016), but the relationship between WBI and negative physical health outcomes is less established (Pearl and Puhl, 2018).

The present study examined the relationship between WBI and QOL specific to weight by using the Impact of Weight on Quality of Life-Lite scale (IWQOL-Lite). The scale provides a total score and values on five subscales: Physical Function, Self-Esteem, Sexual Life, Public Distress, and Work (Kolotkin et al., 2001). A previous study by Hübner et al. investigated the relationship between IWQOL and WBI; however, they only examined the IWQOL-Lite Total score and the Self-Esteem subscale and did not examine the relationship among the four other subscales (Hübner et al., 2016). Evaluating the relationship between WBIS scores and physical functioning, sexual life, public distress, and work would provide a more comprehensive understanding of how WBI may influence QOL. We predicted that greater reports of WBI would be associated with greater impairments in weight-related QOL, as measured by the IWQOL-Lite Total score and all five subscales.

## Materials and Methods

### Study Design

The current study represents a secondary analysis of data from a randomized controlled trial that assessed the efficacy of the weight loss medication lorcaserin, compared with placebo, for maintaining weight loss achieved with a low-calorie diet. The methodology and results of the study, which was approved by the University of Pennsylvania’s Institutional Review Board, have been described previously (Shaw Tronieri et al., 2018). The present study is limited to the analysis of participants’ baseline characteristics pertaining to WBI, weight-specific QOL, and symptoms of depression.

### Participants

Participants were recruited by print, online, and radio advertisements and were eligible to participate in the study if they were 21–65 years of age and had a body mass index (BMI) of ≥33 kg/m^2^ and ≤55 kg/m^2^ (or ≥30 kg/m^2^ with an obesity-related comorbidity). Major exclusion criteria included current major depression; pregnancy or lactation; types 1 or 2 diabetes; use of medications that cause weight loss or gain; and a history of bariatric surgery. Participants deemed eligible based on a phone screen completed an in-person behavioral evaluation (of their eating, activity, and mood) and provided written informed consent. Eligible participants then met with a physician or nurse practitioner who completed a history and physical examination. Participants who remained eligible were enrolled in the program.

### Measures

At the screening visit, height was measured in duplicate using a stadiometer (Veeder-Root, Elizabethtown, NC, United States), and weight was measured using a digital scale (Detecto, model 6800 A). Weight was measured again at week 1 of the intervention, from which baseline BMI was calculated. Participants received questionnaires 2 weeks prior to the first group treatment session to be completed online (via REDcap) or via mail. Questionnaires were completed prior to the first treatment visit. Measures included the Weight Bias Internalization Scale (WBIS), the Patient Health Questionnaire (PHQ-9), and the IWQOL-Lite. The 11-item WBIS evaluates the degree to which people assign weight-based stereotypes to themselves (e.g., “I am less attractive than most other people because of my weight”) and devalue themselves due to weight (e.g., “I hate myself for being overweight”). Participant responses are rated on a scale of 1 *(strongly disagree)* to 7 *(strongly agree)*, with higher scores indicating greater WBI (Durso and Latner, 2008). The American Psychiatric Association recommends the PHQ-9 as an optimal brief screening inventory for assessing symptoms of depression (American Psychiatric Association, 2018). Respondents rate the frequency of symptoms of depression during the previous 2 weeks, using a scale from 0 (not at all) to 3 (nearly every day). The higher the total score, the greater the severity of symptoms of depression. Scores ≥15 reflect severe symptoms of depression. The reliability and validity of the PHQ-9 have been shown to be excellent (American Psychiatric Association, 2018). The 40-item IWQOL-Lite provides an overall measure of QOL as affected by weight (Total score), as well as scores on the five subscales of Physical Function, Self-Esteem, Sexual Life, Work, and Public Distress. Scores are transformed and scored from 0-100, with higher scores representing a higher weight-related QOL. The IWQOL-Lite has excellent reliability (Kolotkin and Crosby, 2002).

### Analytic Plan

Bivariate correlations were computed between the WBIS and the IWQOL-Lite Total score and all IWQOL-Lite subscales (the findings for which are shown in Supplementary Table 1). Linear regression was used to test the effects of WBIS scores on the IWQOL-Lite Total score and each subscale above and beyond the effects of participant gender, race, age, BMI, and depression (PHQ-9) scores. Based on prior evidence of different effects of WBI based on gender and race (Puhl et al., 2018; Boswell and White, 2015) separate hierarchical regression models were constructed with interaction terms between WBIS scores and gender and race (one model for race and another for gender for the IWQOL-Lite Total score and each subscale, with all of the same covariates described above). PHQ-9 scores were transformed with the natural log to meet assumptions of normality, and all continuous predictor variables were centered at their means. Because all participants completed questionnaires at baseline, missing score values can be attributed to participants skipping individual items within scales. Scale scores were prorated for participants with ≤15% missing values for scale items.

## Results

A total of 178 adults with obesity were enrolled in the trial and completed baseline assessments. Participant characteristics have been previously reported (71.3% black, 21.9% white, 87.6% female, mean age = 44.2 ± 11.2 years, mean BMI = 40.9 ± 5.9 kg/m^2^) (Shaw Tronieri et al., 2018). The mean WBIS score at baseline (*n* = 172) was 3.6 ± 1.1, and the mean PHQ-9 score (*n* = 176) was 4.9 ± 4.8, indicating minimal symptoms of depression.

### Regression Analyses

As shown in Table 1, WBIS scores were significantly associated with the IWQOL-Lite Total score and all subscales above and beyond all covariates. The strongest association was between WBIS scores and the Self-Esteem subscale (*β* = -0.70, *p* < 0.001), and the smallest effect size was between WBIS scores and the Physical Function subscale (*β* = -0.18, *p* = 0.019). Table 1 also shows that greater symptoms of depression, as measured by the PHQ-9, were associated with lower IWQOL-Lite Total scores, as well as lower scores on all five subscales (with *β* values ranging from -0.15 to -0.33). Similarly, higher BMIs tended to be associated with lower overall weight-related QOL (i.e., Total score), as well as lower scores on the physical function, public distress, and work subscales (with *β* values ranging from -0.18 to -0.43). The interactions between WBIS scores and gender and WBIS scores and race were not significant in any analysis (*p* values > 0.10, *R^2^* change values ≤0.01; see Supplementary Tables 2, 3).

**Table 1:** Linear regression analyses.

Variable	IWQOL-Lite Total Score			IWQOL-Lite Physical Function			IWQOL-Lite Self-Esteem			IWQOL-Lite Sexual Life			IWQOL-Lite Public Distress			IWQOL-Lite Work		
	B	SE	*β*	B	SE	*β*	B	SE	*β*	B	SE	*β*	B	SE	*β*	B	SE	*β*
WBIS	-8.48	1.06	-**0.50^∗∗∗^**	-3.58	1.51	-**0.18^∗^**	-16.32	1.37	-**0.70^∗∗∗^**	-10.32	1.92	-**0.40^∗∗∗^**	-7.37	1.60	-**0.33^∗∗∗^**	-7.93	1.42	-**0.40^∗∗∗^**
Female	-0.72	3.52	-0.01	-2.32	5.00	-0.03	-3.09	4.54	-0.04	2.62	6.35	0.03	1.29	5.29	0.02	1.81	4.71	0.03
Black	-0.20	2.50	0.01	0.52	3.56	0.01	2.32	3.22	0.04	1.12	4.52	0.02	-1.09	3.76	-0.02	-3.65	3.35	-0.07
Educ	0.71	0.54	0.08	0.86	0.77	0.08	0.59	0.69	0.05	0.93	0.97	0.07	1.08	0.81	0.09	-0.15	0.72	-0.01
Age	-0.20	0.09	-**0.12^∗^**	-0.47	0.13	-**0.24^∗∗∗^**	<0.01	0.12	<0.01	-0.44	0.17	-**0.17^∗∗^**	0.21	0.14	0.09	-0.11	0.13	-0.06
BMI	-0.94	0.18	-**0.28^∗∗∗^**	-1.42	0.26	-**0.37^∗∗∗^**	0.08	0.24	0.02	-0.44	0.33	-0.09	-1.85	0.28	-**0.43^∗∗∗^**	-0.70	0.25	-**0.18^∗∗^**
PHQ-9	-5.99	1.31	-**0.28^∗∗∗^**	-5.60	1.86	-**0.23^∗∗^**	-5.14	1.68	-**0.17^∗∗^**	-8.72	2.36	-**0.27^∗∗∗^**	-4.13	1.97	-**0.15^∗^**	-8.22	1.75	-**0.33^∗∗∗^**


*Note. WBIS, Weight Bias Internalization Scale; Educ, Education; PHQ-9, Patient Health Questionnaire-9; IWQOL, Impact of Weight on Quality of Life. ^∗^p < 0.05, ^∗∗^p ≤ 0.01, ^∗∗∗^p ≤ 0.001 Ns ranged from 161 to 162.*

## Discussion

The results of this study showed that, in treatment-seeking patients with obesity, those who reported higher levels of WBI also reported lower overall weight-related QOL. The present findings are consistent with prior reports of an association between WBI and lower general QOL, as measured by the global mental and physical health-related QOL scores of the SF-36 (Latner et al., 2013, 2014; Pearl et al., 2014), as well as a report of negative associations between WBIS scores and the IWQOL-Lite Total and Self-Esteem subscale scores (Hübner et al., 2016). Our results extend these findings by documenting relationships between WBI and impairments in several domains of function that are specific to individuals with obesity. In addition to predicting lower overall weight-related QOL, WBI was associated with lower weight-related physical function, self-esteem, sexual life, public distress, and work. These associations highlight that there is a negative relationship between WBI and both mental and physical weight-related outcomes.

Consistent with prior studies, we observed that both higher BMI and greater symptoms of depression were associated with lower overall weight-related QOL and lower weight-related physical function, public distress, and work. Higher body weight and symptoms of depression are well known to adversely affect QOL (Kolotkin et al., 2009). Levels of WBI and depression were relatively low for a treatment-seeking sample (Latner et al., 2013; Pearl et al., 2014), which may be due to study exclusion criteria of major depression or the use of anti-depressant medication. Additionally, our sample consisted predominantly of black participants, who have been shown to have lower levels of WBI than white adults with obesity (Puhl et al., 2018), so our findings may not generalize to other weight loss treatment-seeking samples. We found that WBI further added to impairment in weight-related QOL after controlling for the effects of BMI, symptoms of depression, and demographic characteristics. This finding suggests that the relationship between WBI and weight-related QOL is not fully accounted for by higher body weight or depression scores among patients with greater WBI. Clinical interventions may be needed to reduce WBI in persons with obesity to achieve optimal improvements in weight-related QOL and in related subdomains of functioning.

Given that most prior studies in this area of research have been limited to predominantly white participants, the high proportion of black participants represents a strength of the current study. However, the small number of males in this study may limit the generalizability of these results. We did not find that the relationship between WBI and QOL differed by race or by gender in this sample. Further research with larger, diverse samples is needed to replicate these results. This study also cannot determine the causal nature of the association between WBI and weight-related QOL (or between QOL and BMI and symptoms of depression). We believe that WBI likely contributes to reduced weight-related QOL, both overall and in specific domains of functioning. However, longitudinal studies are needed to demonstrate a causal relationship between WBI and impaired QOL.

In summary, high WBI in persons with obesity was associated with greater impairments in overall weight-specific QOL and in weight-related physical function, self-esteem, sexual life, public distress, and work. These relationships were maintained after controlling for BMI and depression, suggesting that WBI has an independent effect on weight-related QOL. This may suggest that treatments targeting WBI would improve weight-related QOL.

## Author Contributions

OW was one of the study coordinators, participated in conceptualizing this specific study, and held primary responsibility for drafting the manuscript. TW was responsible for the conception and design of the trial, participated in conceptualizing this specific study, and participated in editing of the manuscript. JT served as one of the study interventionists, organized the trial database, and participated in editing the manuscript. AC served as a medical monitor for the trial and participated in editing the manuscript. RP served as one of the study interventionists, participated in conceptualizing this specific study, conducted the statistical analyses for this manuscript, and participated in editing the manuscript.

## Conflict of Interest Statement

OW discloses serving as a consultant for Novo Nordisk. TW serves on scientific advisory boards for Novo Nordisk and Weight Watchers International and has received research funding from both organizations and Eisai Co. JT discloses serving as a consultant to Novo Nordisk. AC discloses serving as a consultant and receiving research funding from Shire Pharmaceuticals. RP discloses serving as a consultant for Novo Nordisk and receiving research funding from Weight Watchers International.
