# Supplementary material for: Weight Bias Internalization Is Negatively Associated With Weight-Related Quality of Life in Persons Seeking Weight Loss
Source: Front Psychol. 2018 Dec 17;9:2576. doi: 10.3389/fpsyg.2018.02576 (PMC6304379; doi:10.3389/fpsyg.2018.02576)
Supplement: Supplementary file 1 [file Table_1.DOCX]

Supplementary Table 1. Bivariate correlations

| Variable | IWQOL-Lite  Total Score | IWQOL-Lite Physical Function | IWQOL-Lite  Self-Esteem | IWQOL-Lite  Sexual Life | IWQOL-Lite  Public Distress | IWQOL-Lite Work |
| --- | --- | --- | --- | --- | --- | --- |
| Weight Bias  Internalization Scale | -0.63*** | -0.28*** | -0.76*** | -0.52*** | -0.43*** | -0.52*** |

Note. IWQOL = Impact of Weight on Quality of Life ****p*<0.001; *N*s ranged from 170-171.

Supplementary Table 2. Regression results including interaction between weight bias internalization and gender.

| Variable | IWQOL-Lite Total Score | | | IWQOL-Lite Physical Function | | | IWQOL-Lite Self-Esteem | | | IWQOL-Lite Sexual Life | | | IWQOL-Lite Public Distress | | | IWQOL-Lite Work | | |
| --- | --- | --- | --- | --- | --- | --- | --- | --- | --- | --- | --- | --- | --- | --- | --- | --- | --- | --- |
|  | B | SE | *β* | B | SE | *β* | B | SE | *β* | B | SE | *Β* | B | SE | *β* | B | SE | *β* |
| WBIS | -10.68 | 3.52 | **-0.63**** | -5.02 | 5.01 | -0.26 | -19.91 | 4.53 | **-0.85***** | -10.02 | 6.35 | -0.39 | -10.75 | 5.29 | **-0.48*** | -10.75 | 4.71 | **-0.55*** |
| Female | -10.66 | 15.55 | -0.18 | -8.83 | 22.14 | -0.13 | -19.32 | 20.02 | -0.23 | 3.95 | 28.07 | 0.04 | -13.98 | 23.39 | -0.17 | -10.92 | 20.81 | -0.15 |
| Black | 0.24 | 2.51 | 0.01 | 0.55 | 3.57 | 0.01 | 2.38 | 3.23 | 0.04 | 1.12 | 4.53 | 0.02 | -1.04 | 3.77 | -0.02 | -3.60 | 3.35 | -0.07 |
| Age | -0.21 | 0.10 | **-0.12*** | -0.48 | 0.14 | **-0.24***** | -0.01 | 0.12 | <-0.01 | -0.44 | 0.17 | **-0.17*** | 0.20 | 0.14 | 0.09 | -0.12 | 0.13 | -0.06 |
| Educ | 0.70 | 0.54 | 0.07 | 0.85 | 0.77 | 0.08 | 0.57 | 0.70 | 0.04 | 0.93 | 0.98 | 0.07 | 1.05 | 0.81 | 0.08 | -0.17 | 0.72 | -0.02 |
| BMI | -0.94 | 0.18 | **-0.28***** | -1.42 | 0.26 | **-0.37***** | 0.08 | 0.24 | 0.02 | -0.44 | 0.33 | -0.09 | -1.86 | 0.28 | **-0.43***** | -0.70 | 0.25 | **-0.18**** |
| PHQ-9 | -6.02 | 1.31 | **-0.28***** | -5.63 | 1.87 | **-0.23**** | -5.19 | 1.69 | **-0.18**** | -8.71 | 2.37 | **-0.27***** | -4.18 | 1.97 | **-0.15*** | -8.26 | 1.75 | **-0.33***** |
| WBIS x Female | 2.39 | 3.63 | 0.19 | 1.56 | 5.17 | 0.11 | 3.89 | 4.68 | 0.23 | -0.32 | 6.56 | -0.02 | 3.66 | 5.47 | 0.22 | 3.05 | 4.86 | 0.21 |

Note. WBIS = Weight Bias Internalization Scale; Educ = Education; PHQ-9 = Patient Health Questionnaire – 9; IWQOL = Impact of Weight on Quality of Life **p*<0.05 ***p*<0.01 ****p*≤0.001 *N*s ranged from 161-162.

Supplementary Table 3. Regression results including interaction between weight bias internalization and race

| Variable | IWQOL-Lite Total Score | | | IWQOL-Lite Physical Function | | | IWQOL-Lite Self-Esteem | | | IWQOL-Lite Sexual Life | | | IWQOL-Lite Public Distress | | | IWQOL-Lite Work | | |
| --- | --- | --- | --- | --- | --- | --- | --- | --- | --- | --- | --- | --- | --- | --- | --- | --- | --- | --- |
|  | B | SE | *β* | B | SE | *β* | B | SE | *β* | B | SE | *Β* | B | SE | *β* | B | SE | *β* |
| WBIS | -8.42 | 1.76 | **-0.50***** | -3.59 | 2.51 | -0.18 | -18.14 | 2.26 | **-0.78***** | -12.63 | 3.17 | **-0.49***** | -4.02 | 2.63 | -0.18 | -6.04 | 2.35 | **-0.31*** |
| Female | -0.72 | 3.53 | -0.01 | -2.32 | 5.02 | -0.03 | -3.06 | 4.53 | -0.04 | 2.67 | 6.35 | 0.03 | 1.23 | 5.27 | 0.02 | 1.77 | 4.71 | 0.03 |
| Black | 0.57 | 8.47 | 0.01 | 0.49 | 12.05 | 0.01 | -8.20 | 10.88 | -0.14 | -12.22 | 15.24 | -0.19 | 18.22 | 12.64 | 0.33 | 7.25 | 11.30 | 0.15 |
| Age | -0.20 | 0.09 | **-0.12*** | -0.47 | 0.13 | **-0.24***** | <-0.01 | 0.12 | <-0.01 | -0.45 | 0.17 | **-0.17**** | 0.22 | 0.14 | 0.10 | -0.11 | 0.13 | -0.05 |
| Educ | 0.71 | 0.54 | 0.08 | 0.86 | 0.77 | 0.08 | 0.62 | 0.69 | 0.05 | 0.97 | 0.97 | 0.07 | 1.02 | 0.81 | 0.08 | -0.18 | 0.72 | -0.02 |
| BMI | -0.94 | 0.18 | **-0.28***** | -1.42 | 0.26 | **-0.37***** | 0.11 | 0.24 | 0.02 | -0.42 | 0.33 | -0.08 | -1.89 | 0.28 | **-0.43***** | -0.72 | 0.25 | **-0.19**** |
| PHQ-9 | -5.99 | 1.31 | **-0.28***** | -5.60 | 1.87 | **-0.23**** | -5.21 | 1.69 | **-0.18**** | -8.80 | 2.36 | **-0.27***** | -4.01 | 1.96 | **-0.14*** | -8.15 | 1.75 | **-0.33***** |
| WBIS x Black | -0.09 | 2.07 | -0.01 | 0.01 | 2.95 | <0.01 | 2.70 | 2.67 | 0.18 | 3.43 | 3.74 | 0.21 | -4.95 | 3.10 | -0.35 | -2.79 | 2.77 | -0.22 |

Note. WBIS = Weight Bias Internalization Scale; Educ = Education; PHQ-9 = Patient Health Questionnaire – 9; IWQOL = Impact of Weight on Quality of Life **p*<0.05 ***p*<0.01 ****p*≤0.001 *N*s ranged from 161-162.
